# Associations of PM_2.5_ exposure with diabetes-related mortality for California residents

**DOI:** 10.3389/fpubh.2026.1903823

**Published:** 2026-08-27

**Authors:** Richy Zheng, Jason G. Su, Eahsan Shahriary, Sadeer Al-Kindi, Michael Jerrett, Timothy T. Brown, Rob McConnell, John Balmes

**Affiliations:** 1 School of Public Health, University of California at Berkeley, Berkeley, CA, United States; 2 Houston Methodist & Weill Cornell Medicine, Houston Methodist Research Institute, Houston, TX, United States; 3 Fielding School of Public Health, University of California at Los Angeles, Los Angeles, CA, United States; 4 Keck School of Medicine, University of Southern California, Los Angeles, CA, United States; 5 School of Medicine, University of California at San Francisco, San Francisco, CA, United States

**Keywords:** air pollution, California Department of Public Health, diabetes-related mortality, fine particulate matter, type 2 diabetes

## Abstract

**Background:**

Emerging evidence links air pollution exposure to metabolic dysfunction; however, few studies have examined diabetes-related mortality in relation to ambient air pollutants using high-resolution exposure data at the population level. In the United States, particularly in large and geographically diverse states such as California, exposure contrasts and population heterogeneity provide an important setting to evaluate these associations.

**Methods:**

We conducted a matched case–control analysis using California Department of Public Health (CDPH) Vital Records (2010–2021). Diabetes-related mortality events (ICD-10 E11) were identified as primary or contributory causes. Decedents (cases) were geocoded to residential addresses, and one-year rolling averages of fine particulate matter (PM_2.5_) before death were assigned as individual exposures. Each death record was matched to its selected controls based on month and year of birth and race-ethnicity. Controls were identified from the same statewide CDPH mortality database and were eligible because they had not died by the corresponding case’s date of death. Because the number of eligible controls varied across matched strata, controls were randomly sampled within each matched stratum to achieve an overall control-to-case ratio of approximately 2:1 for the study population. The final dataset included 60,824 diabetes-related deaths and 119,053 controls. Exposures were standardized by their interquartile range (IQR) and conditional logistic regression models estimated associations between 1 year rolling average fine particulate matter (PM_2.5_) exposure and odds of diabetes-related mortality, adjusting for age, sex, race-ethnicity, marital status, and education. Nitrogen dioxide (NO_2_) was included as a co-pollutant for confounding control.

**Results:**

PM_2.5_ exposure (per 2.65 μg/m^3^ IQR increase) was associated with a 18% higher odds of diabetes-related mortality (OR = 1.18; 95% CI: 1.15–1.22) before traffic indicator NO_2_ adjustment and showed a stronger association with 21% higher odds (OR = 1.21; 95% CI: 1.17–1.25) after NO_2_ adjustment. Health economics analysis estimated that reducing PM_2.5_ exposure by its IQR could avoid losses of $31.2 million per 100,000 people.

**Conclusion:**

Higher ambient PM_2.5_ exposure was associated with increased odds of diabetes-related mortality in California even after adjustment for NO_2_ and other impact factors. These findings support the need for continued strengthening of ambient air quality regulations.

## 1 Introduction

Type 2 diabetes (T2D) has emerged as one of the most prevalent and costly chronic diseases worldwide, imposing a growing burden on healthcare systems and communities. Characterized by insulin resistance and impaired glucose regulation, T2D contributes to cardiovascular disease, renal complications, neuropathy, and premature death (1–4). In the United States, diabetes affects over 38 million people, with nearly 95% of cases classified as T2D (5). California, the most populous and environmentally diverse state, bears a particularly heavy burden. Data from the California Health Interview Survey (CHIS) show that approximately 10.8% of adults in the state has been diagnosed with diabetes (see https://healthpolicy.ucla.edu/our-work/health-promotion-disease-prevention-program), with prevalence disproportionately higher among Hispanic, Black, and lower-income populations. Although established risk factors such as age, obesity, diet, physical inactivity, and family history remain central to T2D development, accumulating evidence points to air pollution as an important, modifiable environmental determinant (6–10). Epidemiologic and toxicologic studies over the past two decades have demonstrated that exposure to airborne pollutants, especially fine particulate matter (PM_2.5_), can adversely affect metabolic function (10). A recent prospective cohort study (11) found that specific PM_2.5_ chemical components were associated with dyslipidemia, supporting the hypothesis that particle composition may influence metabolic toxicity beyond total PM_2.5_ mass concentration. These pollutants induce oxidative stress, systemic inflammation, and endothelial dysfunction, which are key pathways in insulin resistance and glucose dysregulation (12, 13). Long-term exposure to PM_2.5_ has been linked to increased diabetes incidence and mortality (10, 14–16).

Although numerous epidemiologic studies have reported associations between PM_2.5_ exposure and diabetes-related outcomes, including diabetes incidence, prevalence, and mortality, important knowledge gaps remain regarding the role of fine-scale spatial and temporally resolved exposure variability in diabetes-related mortality. Many prior studies have relied on relatively coarse-resolution exposure estimates (e.g., satellite-based or chemical transport model products at kilometer-scale resolution) (17–20), or on exposure surfaces based on annual averages or multi-year mean concentrations (20, 21). While these approaches can capture broad regional patterns of air pollution, they may not adequately represent local spatial gradients or temporal variability in PM_2.5_ concentrations. In particular, assigning the same annual or multi-year average concentration to individuals who died on different days may fail to capture meaningful differences in exposure conditions preceding mortality events, potentially introducing exposure misclassification. This limitation may be especially relevant in urban environments where PM_2.5_ concentrations can vary substantially over short distances and time periods due to traffic emissions, meteorological conditions, and other neighborhood-scale factors. Such exposure misclassification can attenuate estimated associations and obscure the potential health impacts of localized pollution exposures. In addition, many existing studies have been conducted in geographically limited populations or specific cohorts, leaving uncertainty regarding whether associations between PM_2.5_ exposure and diabetes-related mortality persist across a large and environmentally diverse region such as California. To address these limitations, the present study leverages newly developed high-resolution air pollution exposure surfaces that provide daily PM_2.5_ concentrations at a 100 m grid resolution across California over more than three decades (1989–2021) (22). These exposure surfaces integrate satellite observations, regulatory ground monitoring data, Google Street View mobile measurements, meteorological information, roadway specific traffic data, land use characteristics and other built-environment characteristics, with most predictors developed at a 30 m spatial resolution, to characterize fine-scale spatial and temporal variability in PM_2.5_ concentrations.

**Figure 1:**
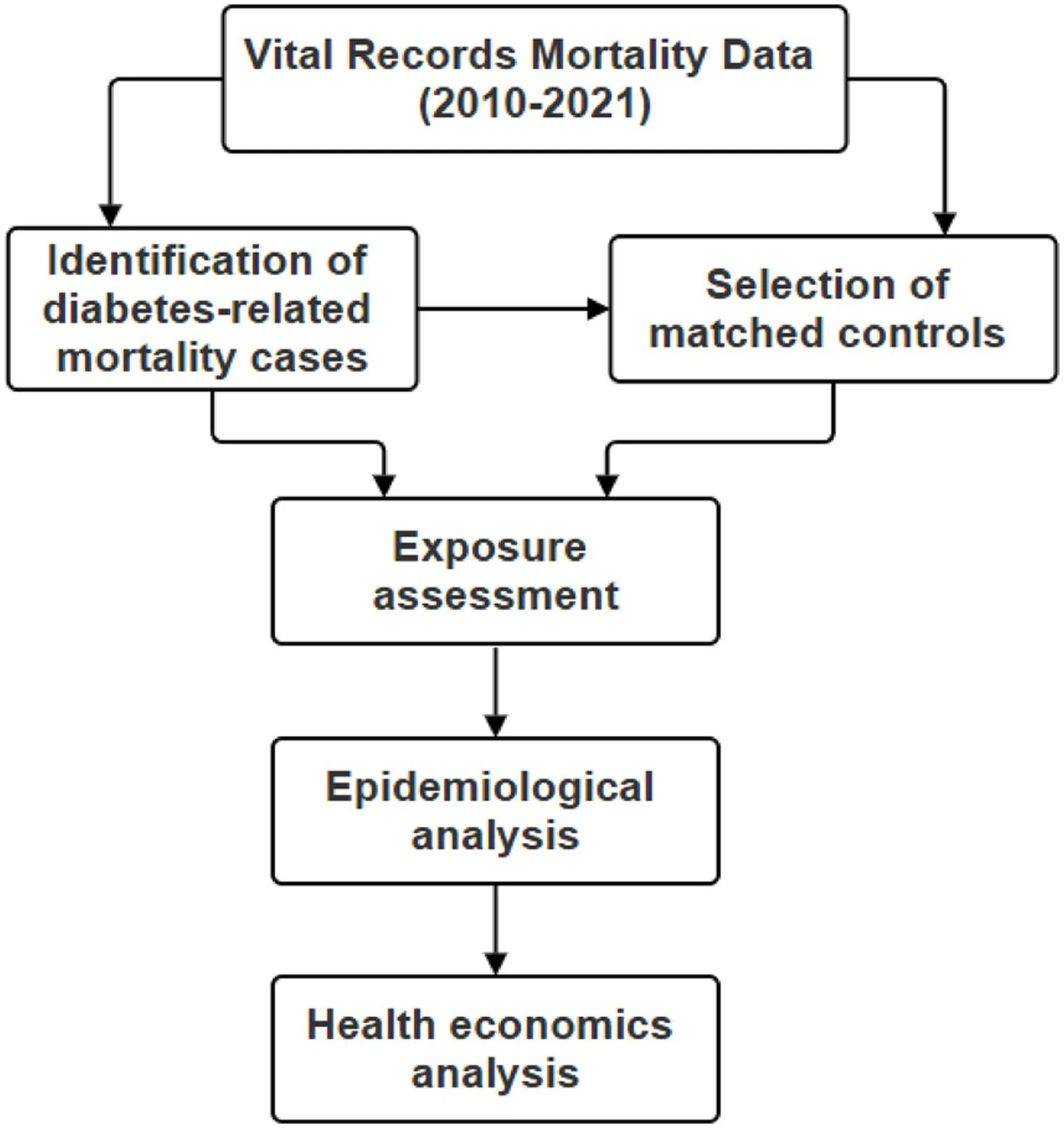
The overall analytical framework of the study, from population selection and exposure assessment to epidemiologic analysis and health economic evaluation.

By combining statewide diabetes mortality records with validated high-resolution PM_2.5_ exposure estimates, this study provides a unique opportunity to evaluate associations between long-term PM_2.5_ exposure and diabetes-related mortality while reducing exposure misclassification associated with coarse-resolution and temporally aggregated exposure models. The findings contribute new evidence regarding the importance of fine-scale exposure characterization in environmental epidemiology and provide insights into spatial variations in diabetes-related mortality risk across diverse California communities. Figure 1 provides an overview of the study design and analytical workflow.

## 2 Materials and methods

### 2.1 Development of PM_2.5_ exposure surfaces

The PM_2.5_ concentrations used in this study were derived from a previously developed land-use regression (LUR) model that estimated daily PM_2.5_ concentrations at a 100 m spatial resolution (22). The model incorporated measured concentrations from regulatory and saturation monitoring networks and multiple spatial predictors, including satellite-derived aerosol optical depth, meteorological conditions, traffic-related variables, land-use characteristics, elevation, and other geographic factors. Importantly, most predictor variables were developed at a 30 m spatial resolution to capture fine-scale environmental characteristics and emission-related factors that influence local PM_2.5_ variability. Model performance was evaluated using a v-fold cross-validation approach. In this procedure, the monitoring dataset was randomly divided into *v* approximately equal subsets (folds). The model was trained using *v-1* folds and then evaluated using the remaining fold, with this process repeated *v* times so that each fold was independently used as a validation dataset. The overall model performance was calculated by aggregating predictions across all validation folds. This approach provides an estimate of how well the model predicts PM_2.5_ concentrations at locations not used during model training and reduces the likelihood that model performance is inflated by evaluating predictions at the same locations used for model development. The v-fold cross-validation results showed an adjusted R^2^ of 0.65 between predicted and measured annual PM_2.5_ concentrations. This corresponds to a correlation coefficient (r) greater than 0.80 between model predictions and observed concentrations, demonstrating strong agreement between estimated and measured PM_2.5_ levels. These validation results provide evidence that the model reliably captures spatial patterns of PM_2.5_ concentrations.

### 2.2 Data acquisition

The University of California at Berkeley (UCB) research team acquired the California Department of Public Health (CDPH) Vital Records for diabetes-related mortality to assess the impacts of air pollution on metabolic health outcomes across California from 2010 to 2021. For the data provided by CDPH, Institutional Review Board (IRB) approval was obtained to ensure the secure and ethical use of human subject data by authorized UCB researchers. We submitted applications to both the UCB Institutional Review Board (for reliance on State Committee for Protection of Human Subjects - CPHS) and the California Health and Human Services Committee, both of which reviewed and approved our research protocol. Following approval, we worked with CDPH to acquire related data under a strict data-use agreement to protect confidentiality and the acquired data were stored on secure UCB Secure Research Data Center (SRDC) servers in compliance with the Health Insurance Portability and Accountability Act (HIPAA) and state requirements.

In this study, diabetes-related mortality was defined as deaths where diabetes mellitus was listed as either the primary underlying cause of death or a contributing cause of death on the death certificate using ICD-10 code E11 (type 2 diabetes mellitus). We selected this definition because diabetes is frequently reported as a contributing cause of death rather than the sole or primary underlying cause, and restricting the outcome definition only to deaths where diabetes was listed as the sole or primary underlying cause would substantially reduce the number of identified events and underestimate the population burden associated with diabetes. Including both primary and contributory diabetes-related deaths provides a more comprehensive assessment of the mortality burden associated with type 2 diabetes in the California population. Because the primary objective of this study was to evaluate the relationship between PM_2.5_ exposure and the overall mortality burden attributable to diabetes, we retained the prespecified definition of diabetes-related mortality. Variables collected from the CDPH Vital Records included residential address, date of death, age, gender, race-ethnicity, smoking status, body mass index (BMI), and insurance information.

### 2.3 Identify concentration-response relationships between air pollution exposures and mortality

#### 2.3.1 Assigning air pollution exposure to locations of subjects

For CDPH mortality data (2010–2021), residential addresses decedents and matched controls were geocoded to obtain precise spatial locations. Daily PM_2.5_ exposures were obtained from previously developed high-resolution (100 m × 100 m) concentration surfaces for California (22). These exposure models were constructed by integrating long-term regulatory monitoring data with machine learning-based data fusion methods, combining multiple data streams including ground-based air quality measurements, satellite-derived aerosol optical depth, meteorological reanalysis fields, land-use/land-cover characteristics, and traffic-related indicators. This approach generates spatially and temporally resolved pollutant estimates that capture both local-scale variability and longer-term trends across the state. In the present study, the residential address of each subject was assigned PM_2.5_ exposure values by linking their geocoded location to the corresponding grid-cell estimates for the relevant exposure period. Specifically, for each decedent (case), a one-year rolling mean of PM_2.5_ concentrations prior to the date of death was calculated and assigned as the individual’s air pollution exposure. For each matched control, the exposure was calculated over the corresponding one-year period preceding the matched case’s date of death (i.e., the index date), ensuring that cases and controls were assigned exposure windows of identical duration and calendar time. Daily nitrogen dioxide (NO_2_) exposure was developed and assigned using the same procedures as daily PM_2.5_ and was included to adjust for potential confounding from co-occurring air pollutants. Each death record was matched to its selected controls based on month and year of birth and race-ethnicity to minimize confounding by age and demographic factors. Controls were identified from the same statewide CDPH mortality database and were eligible because they had not died by the corresponding case’s date of death, although some controls subsequently died later during the study period. In a case–control design, increasing the number of controls can improve statistical precision; however, the incremental gain in statistical power decreases substantially as the number of controls increases, particularly when the pool of eligible controls is large. Because the number of eligible controls varied across matched strata, controls were randomly sampled within each matched stratum to achieve an overall control-to-case ratio of approximately 2:1 for the study population. This approach provided an appropriate balance between improving statistical precision and maintaining a manageable analytical dataset. For these matched controls, the corresponding one-year rolling mean exposures were assigned using the same procedure, based on their geocoded residential addresses and the same temporal exposure windows.

#### 2.3.2 Statistical analysis

For diabetes-related mortality using the CDPH data (2010–2021), race-ethnicity was reclassified into five categories (Non-Hispanic White, Non-Hispanic Black, Non-Hispanic Asian, Hispanic, and Other). Records with less than 1 year of residence in the county were excluded to minimize exposure misclassification. We examined exposure based on the annual (365-day) rolling average concentration of pollutants preceding the date of death. Exposures were standardized by their IQR to facilitate interpretation. Conditional logistic regression models were used to estimate the associations between PM_2.5_ exposures and the odds of mortality. PM_2.5_ exposure was analyzed as continuous variables and standardized based on its interquartile ranges (IQRs). Associations are reported per IQR increase in PM_2.5_ (μg/m^3^) from both single-pollutant models and models additionally adjusted for NO_2_ exposure (ppb). All the models included covariates for age, sex, race-ethnicity, marital status and education level. Although matching was performed on age and race-ethnicity, these variables were retained as covariates to account for potential residual effects associated with the matching process and to improve the robustness of effect estimates. We note that in conditional logistic regression, matching factors without residual variation within matched sets are inherently controlled through the conditioning process and do not contribute additional information to the estimation.

### 2.4 Economic analysis of avoidable diabetes-related mortality

To translate the estimated exposure–response relationships into a policy-relevant economic metric, we computed the avoidable loss in the value of statistical life (VSL) attributable to a one-interquartile-range reduction in long-term ambient PM_2.5_. We followed the potential impact fraction (PIF) framework (23, 24), in which the proportion of cases avoidable under a specified shift in exposure is given by PIF = 1–1/OR_IQR_, under the assumption that OR_IQR_ approximates the corresponding population risk ratio when the outcome is rare (25). The avoidable loss of VSL associated with a one-IQR reduction in pollutant exposure was then calculated as the product of the PIF, the baseline diabetes mortality rate, and the VSL.

Three inputs were used. First, the OR_IQR_ estimates from the matched case–control mortality models described in the Results section (PM_2.5_: OR = 1.21 per 2.65 μg/m^3^ IQR), estimated under conditional logistic regression as is standard for matched case–control designs (26). Second, because the CDPH matched analytic file oversamples cases by design and therefore does not reflect the true population diabetes mortality rate, the population baseline rate was obtained separately from the CDC WONDER Underlying Cause of Death database (https://wonder.cdc.gov/ucd-icd10.html; ICD-10 code E11) and averaged across 2010–2019, yielding 12.4 deaths per 100,000 population per year (age-adjusted). Third, we used the standard Value of a Statistical Life of $14.5 million in 2024 constant U. S. dollars, drawn from the California Air Resources Board valuation guidance based on the South Coast Air Quality Management District mortality risk reduction valuation document.^1^ Variances and 95% confidence intervals for the avoidable VSL estimates were obtained using a variation of the delta method as developed for attributable-risk and related impact-fraction quantities derived from logistic models (27). Statewide aggregates can be obtained by linear scaling of the per-100,000 estimates to the California adult population, and we present the per-100,000 figures as the primary output to make this scaling transparent.

## 3 Results

A total of 60,824 diabetes deaths were included in the analysis (Table 1). The mean age at death was 74.2 years (SD = 13.1). Diabetes mortality was concentrated among older adults, with 76.3% of deaths occurring among individuals aged 65 years or older. The largest age group was 75–84 years, accounting for 27.4% of all diabetes deaths, followed by individuals aged 85 years or older (24.9%) and those aged 65–74 years (24.0%). In contrast, deaths among younger adults were relatively uncommon, with only 1.8% occurring among individuals younger than 45 years and 6.0% among those aged 45–54 years. Males accounted for 54.8% of diabetes deaths, while females represented 45.2%. The racial and ethnic distribution of diabetes deaths was diverse, with Non-Hispanic Whites comprising the largest group (42.7%), followed by Hispanics (30.3%), Non-Hispanic Asians (13.3%), and Non-Hispanic Blacks (10.6%). Individuals classified as Other race/ethnicity accounted for 3.2% of diabetes deaths. Educational attainment varied across decedents. High school graduates or individuals with a GED represented the largest educational category (33.6%), followed by those with less than a high school education (26.6%) and those with some college education but no degree (16.3%). Approximately 22.5% of decedents had completed an associate degree or higher, including 10.8% with a bachelor’s degree, 6.0% with an associate degree, and 5.7% with a graduate degree. Educational attainment was unknown for 1.1% of decedents. Average annual pollutant exposures among diabetes decedents were 10.34 μg/m^3^ (SD = 2.20) for PM2.5 and 10.02 ppb (SD = 3.68) for NO2.

**Table 1:** Diabetes death population descriptive statistics.

Characteristic	Diabetes deaths (*N* = 60,824)	Controls (*N* = 119,053)
Age, mean (SD)	74.2 (13.1)	72.4 (17.9)
Age Group		
0–44	1,080 (1.8%)	9,233 (7.8%)
45–54	3,641 (6.0%)	9,115 (7.7%)
55–64	9,753 (16.0%)	16,893 (14.2%)
65–74	14,579 (24.0%)	21,383 (18.0%)
75–84	16,648 (27.4%)	26,915 (22.6%)
85+	15,123 (24.9%)	35,514 (29.8%)
Sex		
F	27,509 (45.2%)	58,033 (48.7%)
M	33,315 (54.8%)	61,020 (51.3%)
Race/Ethnicity		
Non-Hispanic White	25,957 (42.7%)	31,160 (26.2%)
Non-Hispanic Black	6,439 (10.6%)	22,677 (19.0%)
Non-Hispanic Asian	8,100 (13.3%)	24,818 (20.8%)
Hispanics	18,408 (30.3%)	30,289 (25.4%)
Other	1920 (3.2%)	10,109 (8.5%)
Education		
< High School	16,151 (26.6%)	28,547 (24.0%)
High School/GED	20,408 (33.6%)	39,422 (33.1%)
Some College	9,896 (16.3%)	17,972 (15.1%)
Associate Degree	3,665 (6.0%)	7,714 (6.5%)
Bachelor’s Degree	6,548 (10.8%)	15,196 (12.8%)
Graduate Degree	3,468 (5.7%)	8,294 (7.0%)
Unknown	688 (1.1%)	1908 (1.6%)
PM_2.5_, mean (SD)	10.34 (2.20)	10.09 (2.05)
NO_2_, mean (SD)	10.02 (3.68)	10.31 (4.09)

**Figure 2:**
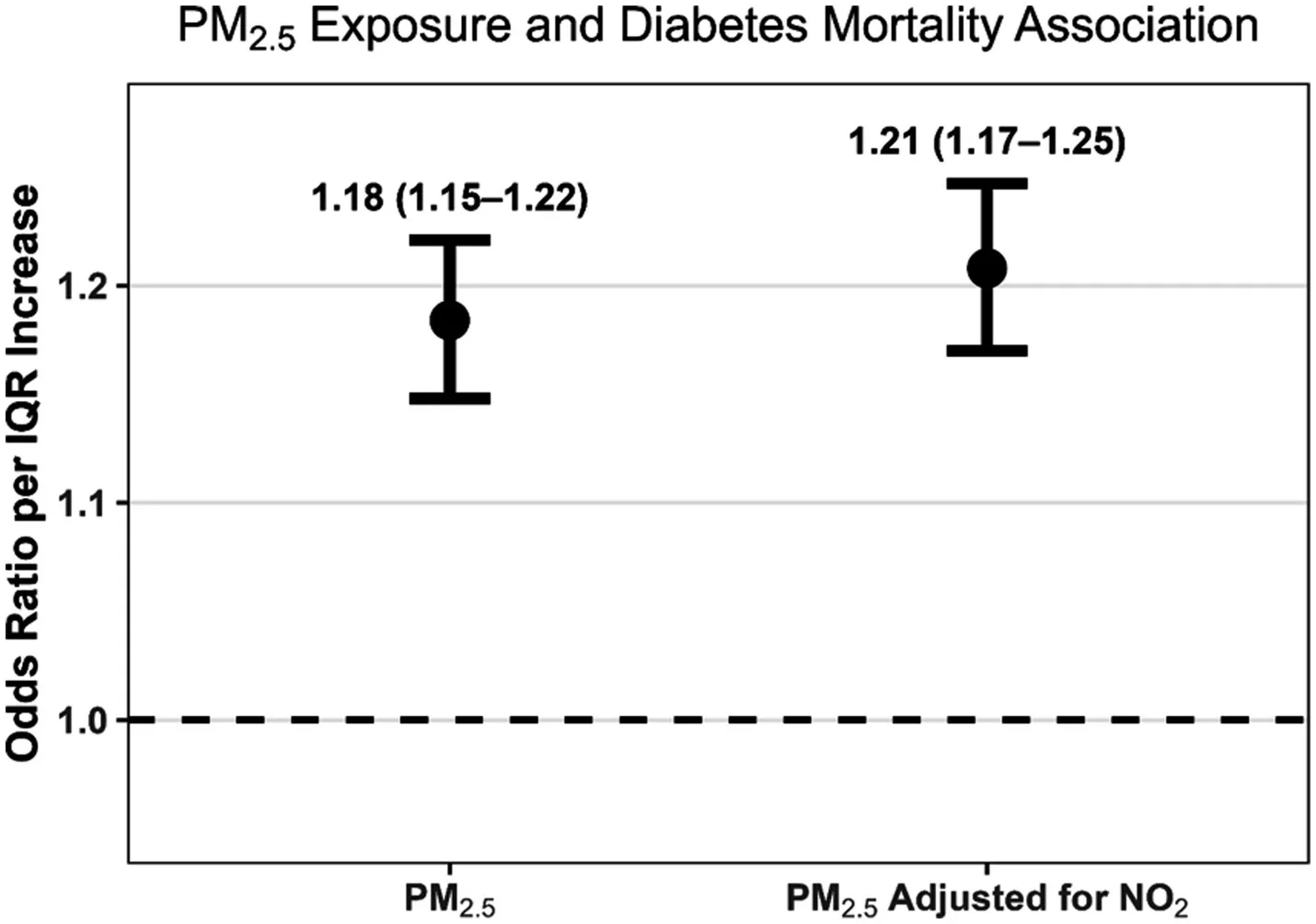
Association of PM_2.5_ exposure (standardized with an IQR of 2.65 μg/m^3^) with diabetes mortality in single- and two-pollutant models.

Mortality analyses using CDPH data from 2010–2021 revealed statistically significant positive associations between 1 year rolling average PM_2.5_ exposure and odds of diabetes-related mortality among California residents (Figure 2). In the single-pollutant model, each 2.65 μg/m^3^ IQR increase in PM_2.5_ exposure was associated with 18% higher odds of death (OR = 1.18; 95% CI: 1.15–1.22). This estimate indicates a robust relationship between annual average fine particulate exposure and mortality risk. To evaluate the potential influence of traffic-related air pollution, NO_2_ was included as a co-pollutant for confounding control. After adjustment for NO_2_, the association between PM_2.5_ and diabetes mortality remained statistically significant and became slightly stronger, with each 2.65 μg/m^3^ IQR increase in PM_2.5_ associated with 21% higher odds of death (OR = 1.21; 95% CI: 1.17–1.25). The modest strengthening of the PM_2.5_ association after adjustment for NO_2_ suggests that the observed relationship is not explained by confounding from traffic-related pollution. The larger magnitude of the PM_2.5_ effect support the hypothesis that combustion-related fine particles and secondary aerosols may play an important role in pathways leading to metabolic dysregulation, systemic inflammation, and cardiovascular complications that ultimately contribute to diabetes-related death.

Across both models, PM_2.5_ exhibited consistently elevated and statistically significant associations with diabetes mortality. These findings align with prior epidemiologic evidence linking fine particulate exposure to cardiometabolic morbidity and mortality. Overall, the results highlight PM_2.5_ as a key environmental determinant of diabetes-related mortality in California, with the association remaining robust after adjustment for NO_2_.

Translating these mortality associations into avoidable economic loss using the PIF framework, the baseline California diabetes mortality rate of 12.4 per 100,000 per year, and a Value of a Statistical Life of $14.5 million (2024 USD) yielded the following estimates per one-IQR reduction in long-term pollutant exposure. For PM_2.5_ (PIF = 0.174), the corresponding avoidable VSL was $31.204 million per 100,000 population (95% CI: $26.291–$36.119 million) for a 2.65 μg/m^3^ reduction. The substantially larger PM_2.5_ figure reflects both the larger odds ratio per IQR and the tighter confidence bounds around that estimate. Linear scaling of these per-100,000 figures to California’s adult population implies aggregate avoidable losses on the order of several billion dollars annually, although such state-level totals should be interpreted as approximations rather than precise estimates.

## 4 Discussion

Analyses using CDPH mortality data revealed that PM_2.5_ was significantly associated with increased odds of T2D-related deaths, reinforcing the health burden of chronic air pollution exposure. The observed associations are consistent in direction across pollutants and persist after adjustment for key demographic and socioeconomic covariates, suggesting robustness of the exposure–response relationship in a large, statewide population over an extended study period (2010–2021). This pattern is biologically plausible given the physicochemical properties of fine particulate matter, including its small aerodynamic diameter and high surface area, which facilitate deep pulmonary deposition. PM_2.5_ exposure has been shown to disrupt autonomic nervous system function and induce persistent low-grade inflammation and oxidative stress, providing plausible biological pathways linking air pollution to insulin resistance, impaired glucose metabolism, and diabetes progression. Recent studies (28) further suggest that transient receptor potential (TRP) channels, particularly TRPV1, may function as molecular sensors of particulate pollutants and oxidative stress, modulating neurogenic inflammation, autonomic regulation, and systemic glycemic control. Although the specific contribution of these pathways to diabetes-related mortality requires further investigation, they provide additional biological plausibility for the associations observed in the present study. Furthermore, T2D should be viewed as a progressive, multi-organ disease rather than a disorder confined to glucose metabolism. As the disease progresses, chronic metabolic dysregulation contributes to cumulative injury across multiple organ systems, including the cardiovascular, renal, nervous, and microvascular systems, ultimately increasing the risk of premature mortality. Emerging trajectory-based frameworks further emphasize that T2D progression involves interconnected transitions among multiple organ systems rather than isolated complications developing independently (29). Within this broader framework, chronic exposure to ambient PM_2.5_ may contribute not only to impaired glucose regulation but also to systemic inflammation, oxidative stress, endothelial dysfunction, and vascular injury that accelerate the progression of diabetes-related complications across multiple organs. These mechanisms provide a coherent framework linking long-term exposure to cardiometabolic dysregulation and increased mortality risk. These findings are broadly consistent with prior epidemiologic evidence demonstrating associations between ambient particulate matter exposure and all-cause, cardiovascular, and metabolic mortality outcomes (30–33).

To address socioeconomic confounding, the study incorporated education as a key covariate in the analytical models. Although individual-level smoking status is an important potential confounder in air pollution epidemiology, smoking information was not available for all individuals in the CDPH Vital Records dataset used in this study. Educational attainment is widely used as an indicator of socioeconomic position in epidemiologic studies because it captures multiple dimensions of social advantage, including health knowledge, employment opportunities, access to resources, and health-related behaviors (34). Education is also strongly associated with smoking patterns and other lifestyle-related risk factors that influence chronic disease and mortality (35, 36). Therefore, education has commonly been incorporated as an adjustment variable in environmental epidemiology studies evaluating long-term air pollution exposure and mortality outcomes (37, 38). Nevertheless, we acknowledge that education may not fully account for individual smoking history, and residual confounding due to unmeasured smoking behaviors cannot be completely excluded, even after adjustment for education.

Regarding spatial confounding, we used high spatial resolution (100 m) PM_2.5_ exposure estimates to better characterize local scale exposure variability and reduce exposure misclassification associated with assigning uniform regional exposure levels to heterogeneous communities. The exposure model incorporated fine scale spatial predictors, including 30 m resolution traffic-related characteristics, land-use variables, and built environment factors, which allowed characterization of neighborhood-level pollution gradients. Fine-scale differences in PM_2.5_ concentrations can arise from local emission sources, including traffic-related pollution, land-use patterns, and neighborhood-scale environmental characteristics, which may not be adequately represented by coarser regional exposure models. Although additional regional-scale predictors could potentially increase overall prediction performance, we intentionally did not incorporate regional predictors solely for the purpose of maximizing model R^2^. Such predictors may improve statistical prediction while reducing the ability of the model to resolve localized spatial gradients that are important for evaluating community-level exposure differences and environmental health disparities. Our modeling strategy therefore prioritized preserving fine-scale spatial variability while maintaining robust predictive performance. The validity of the exposure estimates is supported by multiple lines of evidence: (1) integration of measurements from extensive monitoring networks, including more than 330 regulatory monitoring sites and Google Street View mobile measurements in major California metropolitan areas; (2) use of predominantly 30-m resolution predictors representing fine-scale exposure determinants; and (3) independent v-fold cross-validation demonstrating strong agreement between predicted and measured PM_2.5_ concentrations. Together, these results support the suitability of the 100-m PM_2.5_ exposure estimates for epidemiologic analyses examining spatial variations in diabetes mortality risk. To our knowledge, this represents the highest spatial resolution achieved for a statewide, daily PM_2.5_ exposure averaged assessment used in an epidemiologic study, providing unprecedented characterization of within-community exposure variability across California. Furthermore, PM_2.5_ exposure estimates were assigned based on residential geolocation rather than aggregated geographic units such as census tracts, ZIP codes, or administrative regions. This residential-level assignment, combined with the 100 m exposure surfaces, substantially reduces spatial exposure misclassification and improves the accuracy of individual-level exposure assessment. While high spatiotemporal resolution exposure modeling cannot eliminate residual spatial confounding, it minimizes exposure measurement error and improves the ability to evaluate health associations associated with local scale differences in PM_2.5_ concentrations.

In our study, we did not perform additional analyses using 3-year or 5-year exposure windows because the primary objective of this study was to evaluate associations with recent long-term exposure (1 year rolling average) preceding death using temporally resolved daily exposure estimates. Longer averaging periods would substantially smooth temporal variability and could assign similar exposure values to individuals experiencing mortality events several years apart, thereby reducing the temporal specificity of the exposure assessment. Moreover, the comparison of alternative exposure windows was beyond the scope of the present study, which focuses on demonstrating the application and epidemiologic utility of the newly developed daily 100 m PM_2.5_ exposure surfaces.

From a policy and public health perspective, these findings emphasize the importance of continued air quality improvement efforts, especially targeting reductions in fine particulate matter. Given the persistence of T2D as a major public health challenge, even modest pollutant-related increases in risk translate into substantial population-level impacts. These findings support the potential health co-benefits of more stringent air quality standards and targeted emissions reductions in high-exposure communities.

The economic valuation places these mortality findings in policy-relevant terms. Even after acknowledging the wide confidence intervals around these estimates, the magnitudes are large enough to be policy-relevant: the per-IQR figure for PM_2.5_ alone scales to billions of dollars in avoidable mortality cost when extended across California’s adult population. These figures cover only the diabetes-related mortality channel; they do not include avoided medical expenditures or morbidity-related VSL, both of which would compound the total societal benefit of pollutant reductions.

Several methodological caveats apply to the economic valuation. The PIF formulation used here, PIF = 1–1/OR_IQR_, is fundamentally a risk-based and population-level quantity, but the input we supply to it is a conditional odds ratio estimated within matched case–control sets (26). The conditional odds ratio approximates the population risk ratio only when the outcome is rare (25) and when the assumption of collapsibility is reasonable; the odds ratio is known to be non-collapsible, so conditional and marginal effect estimates can differ even in the absence of confounding (39). For diabetes-related mortality in California, the true population annual incidence, obtained from CDC WONDER aggregate data rather than from the CDPH matched analytic file, is approximately 20.07 per 100,000, satisfying the rare-outcome condition at the population level; however, in the matched analytic dataset cases are oversampled by design, so the rare-outcome justification rests on the population baseline rather than on the analytic-file prevalence. We assume that the conditional odds ratio is a reasonable approximation to the population-level risk ratio for purposes of computing avoidable mortality, but readers should treat the resulting avoidable VSL estimates as an approximation that is sensitive to this mapping (24). Other standard caveats also apply: the IQR of the pollutant distribution is itself non-stationary across the study period, the single-pollutant specification cannot rule out residual confounding by co-pollutants, and the linear scaling of per-100,000 estimates to statewide aggregates assumes that pollutant exposure and underlying diabetes mortality risk are distributed uniformly enough across California for such scaling to be informative.

## 5 Conclusion

Mortality analyses further demonstrated that higher annual exposures to PM_2.5_ in the year preceding death were associated with increased diabetes-related mortality, supporting the role of air pollution exposure in disease progression and fatal outcomes. These findings are consistent with a substantial body of epidemiologic and mechanistic evidence linking particulate matter pollution to chronic systemic inflammation, insulin resistance, vascular dysfunction, and broader cardiometabolic stress. The stronger associations observed for PM_2.5_ further underscore its importance as a key driver of population-level health burden, likely reflecting its ability to penetrate deep into the respiratory system and induce systemic biological effects.

From a public health perspective, these results reinforce air pollution as a modifiable environmental determinant of diabetes-related mortality, with implications that extend beyond individual risk to population-level disease burden. Given the high prevalence of type 2 diabetes and its contribution to mortality in California, even modest reductions in exposure could yield meaningful health benefits at scale. These findings support continued strengthening of ambient air quality standards and targeted mitigation of fine particulate and traffic-related emissions, particularly in high-exposure communities.

Overall, this study adds to growing evidence that improving air quality may serve as an important upstream intervention for reducing diabetes-related mortality and advancing cardiometabolic health equity.

## Data Availability

The datasets presented in this article are not readily available because the dataset is accessed under a data use agreement with the California Department of Public Health (CDPH) and approval from the State Committee for the Protection of Human Subjects (CPHS). Individual-level data are restricted to authorized users, may not be redistributed, and re-identification of individuals is strictly prohibited. Data are used solely for approved research purposes and stored on secure, access-controlled systems. Researchers interested in accessing similar data may do so by submitting an application for a data use agreement to CDPH and obtaining approval from CPHS. Requests to access the datasets should be directed to California State CPHS: CPHS@chhs.ca.gov and CDPH Vital Records: VitalRecordsHelp@cdph.ca.gov.

## Footnotes

1
https://ww2.arb.ca.gov/sites/default/files/2021-10/SCAQMD%20Mortality%20Risk%20Reduction%20Valuation.pdf
